# *Eimeria tenella* Translation Initiation Factor eIF-5A That Interacts With Calcium-Dependent Protein Kinase 4 Is Involved in Host Cell Invasion

**DOI:** 10.3389/fcimb.2020.602049

**Published:** 2021-01-22

**Authors:** Shanshan Liang, Hui Dong, Shunhai Zhu, Qiping Zhao, Bing Huang, Yu Yu, Qingjie Wang, Haixia Wang, Shuilan Yu, Hongyu Han

**Affiliations:** ^1^ Key Laboratory of Animal Parasitology of Ministry of Agriculture, Shanghai Veterinary Research Institute, Chinese Academy of Agricultural Sciences, Shanghai, China; ^2^College of Life Sciences, Shanghai Normal University, Shanghai, China

**Keywords:** *Eimeria tenella*, calcium-dependent protein kinase 4, interacting proteins, translation initiation factor eIF-5A, invasion

## Abstract

*Eimeria tenella* is an apicomplexan, parasitic protozoan known to infect poultry worldwide. An important calcium-dependent protein kinase (CDPK) has been identified in plants, green algae, ciliates and apicomplexan, such as *E. tenella*. CDPKs are effector molecules involved in calcium signaling pathways, which control important physiological processes such as gliding motility, reproduction, and host cell invasion. Given that CDPKs are not found in the host, studying the functions of CDPKs in *E. tenella* may serve as a basis for developing new therapeutic drugs and vaccines. To assess the function of CDPK4 in *E. tenella* (*Et*CDPK4), a putative interactor, translation initiation factor eIF-5A (*Et*eIF-5A), was screened by both co-immunoprecipitation (co-IP) and His pull-down assays followed by mass spectrometry. The interaction between *Et*eIF-5A and *Et*CDPK4 was determined by bimolecular fluorescence complementation (BiFC), GST pull-down, and co-IP. The molecular characteristics of *Et*eIF-5A were then analyzed. Quantitative real-time polymerase chain reaction and western blotting were used to determine the transcription and protein levels of *Et*eIF-5A in the different developmental stages of *E. tenella*. The results showed that the transcription level of *Et*eIF-5A mRNA was highest in second-generation merozoites, and the protein expression level was highest in unsporulated oocysts. Indirect immunofluorescence showed that the *Et*eIF-5A protein was found throughout the cytoplasm of sporozoites, but not in the refractile body. As the invasion of DF-1 cells progressed, *Et*eIF-5A fluorescence intensity increased in trophozoites, decreased in immature schizonts, and increased in mature schizonts. The secretion assay results, analyzed by western blotting, indicated that *Et*eIF-5A was a secreted protein but not from micronemes. The results of invasion inhibition assays showed that rabbit anti-r*Et*eIF-5A polyclonal antibodies effectively inhibited cell invasion by sporozoites, with an inhibition rate of 48%.

## Introduction

Coccidiosis is a protozoan disease that damages the intestine of chickens. The disease causes severe economic loss worldwide, and treatment costs account for approximately 30% of total costs (Tardieux and Baum, 2016). Clinical signs of infection include weight loss, reduced weight gain, diarrhea, apathy, and intestinal inflammation (Tomczuk et al., 2015). Coccidiosis is caused by several *Eimeria* species, of which *E. tenella* is the most pathogenic (Wallach et al., 2008; Mengistu et al., 2020). *Eimeria* species have a complex life cycle initiated in the intestinal epithelium of chickens (Wang et al., 2016). Infection begins with the ingestion of sporulated oocysts present in poultry litter (Tabares et al., 2004).

Calcium is a second messenger involved in many intracellular events (Ding et al., 2020). Changes in calcium ion concentration regulates downstream effector molecules in apicomplexan protozoa and play an important role in protein secretion, as well as parasite motility, differentiation, invasion, and egress from host cells (Billker et al., 2009). Calcium-dependent protein kinases (CDPKs) are serine/threonine protein kinases that regulate calcium signaling. CDPK activity is regulated by calcium, but not by calmodulin (CAMs) nor phospholipid effector pathways (Harper et al., 1991). These kinases have been identified in plants (Harper et al., 1991), green algae (Yuasa and Muto, 1992) and protozoa, but not in yeast, nematodes, fruit flies, or mammals (Chang, 2010). In apicomplexans, CDPKs are important effector molecules in calcium signaling pathways and function to control important physiological events. For instance, *Plasmodium* CDPKs are attractive drug targets that are involved in parasite development and transmission as well as host cell invasion (Vidadala et al., 2014). It has been reported that *Plasmodium falciparum* CDPK1 (*Pf*CDPK1) helps regulate parasite motility, zygote development and transmission (Kato et al., 2008; Sebastian et al., 2012). *P. falciparum* CDPK5 (*Pf*CDPK5) is implicated in malaria parasite egress from erythrocytes, whereas *P. berghei* CDPK3 (*Pb*CDPK3) is involved in ookinete gliding motility and invasion of the mosquito midgut (Siden-Kiamos et al., 2006; Dvorin et al., 2010). Genetic experiments have demonstrated that *P. berghei* CDPK4 (*Pb*CDPK4) is essential for microgamete (male gametocytes) exflagellation and sexual maturation in the mosquito (Billker et al., 2004). In *Toxoplasma gondii*, CDPKs are involved in gliding motility, host cell invasion and egress, and other critical developmental processes (Foroutan and Ghaffarifar, 2018). *Tg*CDPK1 seems to play a central role in regulating parasite motility and host cell invasion (Dobrowolski et al., 1997). *Tg*CDPK7 helps maintain the partitioning and number of centrosomes during parasite division in *T. gondii* and is crucial for parasite survival during replication (Morlon-Guyot et al., 2014).

*E. tenella* CDPKs regulate host cell invasion by sporozoites. Invasion was significantly inhibited in sporozoites treated with a rabbit antiserum against *Et*CDPK3 (Han et al., 2013). Furthermore, polyclonal antibodies against recombinant *E. tenella* CDPK4 (r*Et*CDPK4) inhibited cell invasion by approximately 52%. Indirect immunofluorescence assays evaluated *Et*CDPK4 distribution during *E. tenella* development after sporozoite invasion of DF-1 cells *in vitro*. The results suggest that *Et*CDPK4 may be involved in host cell invasion and sporozoite development (Wang et al., 2016). However, the function of *Et*CDPK4 in *E. tenella* remains unclear.

Proteins are the basis of all cellular activities (De Las Rivas and Fontanillo, 2012). Many biological functions are mediated by protein-protein interactions (Peng et al., 2017). Several methods are currently used to study protein interactions, including pull-down and co-immunoprecipitation (co-IP) assays. The pull-down method can detect protein interactions *in vitro* and has become indispensable for analyzing protein interactions due to high specificity (Louche et al., 2017). Co-IP is used to identify protein interactions *in vivo* and is based on the specific binding of antigens to antibodies. This method is widely used to study pathogen-host interactions (Lin and Lai, 2017).

In this study, putative interactors of *Et*CDPK4 were screened by co-IP and His pull-down assays. Results were confirmed by glutathione S-transferase (GST) pull-down, co-IP, and bimolecular fluorescence complementation (BiFC). The characteristics of interacting proteins were determined. The results highlight the importance of CDPK4 and its interactors in *E. tenella* biology and suggest that this molecule may serve as a target for developing transmission-blocking drugs.

## Methods

### Ethics Statement

The animal studies were approved by the Animal Care and Use Committee of the Shanghai Veterinary Research Institute of the Chinese Academy of Agricultural Sciences.

### Parasite Collection and Purification

The Shanghai strain of *E. tenella* was isolated from a poultry farm in Shanghai, China, in 1985, and was maintained in our laboratory (Resource Number: CAAS21111601, Shanghai Veterinary Research Institute, Chinese Academy of Agricultural Sciences) (Huang et al., 1993). The parasites were maintained and propagated in coccidia-free, 2-week-old chickens, as previously described (Tomley, 1997). Unsporulated oocysts (UO) were collected from the ceca of infected chickens 7 days post-infection (pi). Sporulated oocysts (SO) were cultured in 2% potassium dichromate and collected and purified when more than 90% of oocysts had sporulated. Sporozoites (Spz) were purified from SO during *in vitro* excystation (Han et al., 2010). Second-generation merozoites (Mrz) were isolated from the ceca of infected chickens at 115 h postinoculation, as described previously (Zhou et al., 2010). All parasites were stored in liquid nitrogen.

The chicken embryo fibroblast cell line DF-1, derived from East Lansing Line (ELL-0) chicken embryos (Jiang et al., 2012b), was used in invasion inhibition and immunofluorescence assays. In BiFC assays, human embryonic kidney 293T cells were used in transfection experiments (Nakano et al., 2014).

### Co-IP Assays

For screening for proteins potentially interacting with *Et*CDPK4, the anti-r*Et*CDPK4 IgG was purified by using A/G beads (Beyotime, Haimen, China) from 4 ml rabbit anti-r*Et*CDPK4 serum that were prepared in our Lab (Wang et al., 2016) as the bait protein. The 50 μl purified SO were obtained by using 300 μl RIPA lysis buffer (Beyotime) and 3 μl protease inhibitor cocktail (Sigma-Aldrich, St. Louis, USA) as the prey protein.

Fifty micrograms rabbit anti-r*Et*CDPK4 IgG was bound to the A/G resin, and 50 μg whole proteins from SO were immunoprecipitated with IgG at 4°C overnight according to Pierce™ Co-Immunoprecipitation Kit (Thermo Fisher Scientific™, Waltham, MA) instructions. Samples were eluted with elution buffer. The negative control resin provided in the kit was used in the assays. Proteins were separated by SDS-PAGE and silver stained according to the instructions and reagents provided by Beyotime Biotechnology and identified by mass spectrometry.

To assess the interactions between *Et*eIF-5A and *Et*CDPK4, rabbit 50 μg anti-r*Et*CDPK4 IgG was bound to the A/G resin, and both 50 μg r*Et*eIF-5A and 50 μg r*Et*CDPK4 were jointly incubated with the resin at 4°C overnight. Samples were eluted with the elution buffer provided in the kit. PBS was used as the blank control. Negative control was used the negative resin provided in the Kit, other steps were the same as the sample group. Samples were analyzed by SDS-PAGE and western blot, and interacting proteins were screened with anti-r*Et*CDPK4 and anti-r*Et*eIF-5A polyclonal antibodies. The negative control was incubated with the same antibody.

### Pull-Down Assays

His pull-down was used for screening the interactors of *Et*CDPK4. The assays were performed according to the Pierce™ His Protein Interaction Pull-Down Kit (Thermo Scientific™) instructions. 150 μg His-r*Et*CDPK4 (prey) and 450 μg SO proteins (bait) were incubated with the resin overnight. All steps were performed at 4°C. Hexa His (Sangon Biotech, Shanghai, China) was used as the negative control. The samples were separated by SDS-PAGE and silver stained by using the Fast Silver Stain Kit (Beyotime) according to the instructions. Proteins were identified by mass spectrometry.

The interaction between *Et*eIF-5A and *Et*CDPK4 was assessed using a GST pull-down assay according to Pierce™ GST Protein Interaction Pull-Down Kit (Thermo Scientific™) instructions. 150 μg r*Et*CDPK4 (prey) was incubated with 150 μg r*Et*eIF-5A (bait) expressed in soluble form (see detailed steps below) (Wang et al., 2016). The GST-tagged r*Et*eIF-5A was immobilized to glutathione agarose beads. r*Et*CDPK4 was incubated with the bait at 4°C overnight and eluted using elution buffer. GST-tag instead of r*Et*eIF-5A and incubated with glutathione agarose beads was used as negative control. PBS with r*Et*eIF-5A loaded in an empty glutathione-agarose column was used as a blank control to exclude the influence of other non-experimental factors. The samples were resolved on 12% SDS-PAGE and analyzed by western blotting, as described above.

### BiFC Assays

The BiFC assay was used to confirm the interaction between *Et*CDPK4 and *Et*eIF-5A in living cells. The open reading frames (ORFs) of *Et*eIF-5A without stop codons were amplified from the first-strand cDNA of SO with primers containing *EcoR*I and *Xho*I restriction sites (**Table 1**). The first-strand cDNA of SO was prepared according to the molecular cloning procedure. pBiFC-VN155-*Et*eIF-5A was constructed by ligating the fragment into the *EcoR*I and *Xho*I-digested pBiFC-VN155 vector. pBiFC-VC155-*Et*CDPK4 was constructed previously in our laboratory (Lv et al., 2018). Before the BiFC assay, the expression of the recombinant protein was confirmed by immunofluorescence. Before transfection, 293T cells (5.0 × 10^5^ per well) were cultured on a 6-well plate for 16 h. 1.25 μg pBiFC-VN155-*Et*eIF-5A or pBiFC-VC155-*Et*CDPK4 was transfected to 293T cells according to the FuGENE™ HD Transfection Reagent (Promega, Madison, WI, USA) instruction manual, respectively. The plate was incubated at 37°C for 48 h. The 293T cells transfected with pBiFC-VN155-*Et*eIF-5A or pBiFC-VC155-*Et*CDPK4 were fixed in 2% paraformaldehyde in phosphate-buffered saline (PBS) and placed in 1% Triton X-100 in PBS for 15 min, then blocked with PBS containing 2% (w/v) bovine serum albumin for 2 h at 4°C. A rabbit anti-r*Et*eIF-5A antibody (1:100) or rabbit anti-r*Et*CDPK4 antibody (1:100) was added and incubated for 1 h at 37°C respectively. Then the fluorescein isothiocyanate (FITC)-conjugated goat anti-rabbit IgG antibody (1:500, Sigma-Aldrich) was added and incubated. The cell nuclei were stained by incubation in 10 μg/ml 4′,6-diamidino-2-phenylindole (Beyotime) at room temperature for 10 min. The cells were observed under fluorescence microscope (Olympus, Tokyo, Japan). pBiFC-VN155-*Et*eIF-5A and pBiFC-VC155-*Et*CDPK4 were co-transfected using the steps above. pBiFC-bfosVC155 and pBiFC-bjunVN155 were used as positive controls, and pBiFC-bfosVC155 (delta ZIP) and pBiFC-bjunVN155 were used as negative controls. pBiFC-VC155-*Et*CDPK4 and pBiFC-VN155, pBiFC-VC155, and pBiFC-VN155-*Et*eIF-5A were also used as blank controls.

**Table 1:** Sequences of primers used in this study.

Primer ID	Primer sequence
*Et*eIF-5A sense	5’-GCGAATTCGGGCCACCATGTCGGACGCGGAAGAGGTGAG-3’
*Et*eIF-5A antisense	5’-GCCC TCG AGA GAG CTC CTT GCA GGC GAC GAT CT-3’
*Et*eIF-5A-RT sense	5’-CTGCTGGACAACGGCGACTTG-3’
*Et*eIF-5A-RT antisense	5’-CGCTGACGAGAACGCCCTTG-3’
18S rRNA sense	5’-TGTAGTGGAGTCTTGGTGATTC-3’
18S rRNA antisense	5’-CCTGCTGCCTTCCTTAGATG-3’

### Molecular Cloning

Total RNA was extracted from SO with TRIzol Reagent (Invitrogen, CA, USA). SuperScript™ III reverse transcriptase kit (Invitrogen), and oligo-dT primers were used to reverse transcribe RNA into cDNA. Briefly, approximately 100 mg of purified SO were resuspended in 500 μl TRIzol and homogenized by vortexing with an equal volume of glass beads (710-1,180μm, sigma-Aldrich), and shaken together for 2 min and for 30 s in ice, and repeated the step until oocysts were completely broken. Then total RNA was purified from the homogenized supernatant after centrifugation according to manufacturer’s instructions. The quality of total RNA was carried out using a UV spectrophotometer at 260 nm (Eppendorf, Hamburg, Germany). The first-strand cDNA was synthesized from the total RNA with SuperScript™ III Reverse Transcriptase kit as manufacturer’s instructions.

The ORF sequence of *Et*eIF-5A (Gene ID: ETH_00015190) was amplified using primers containing *EcoR*I and *Xho*I restriction sites with the cDNA of SO as a template (**Supplementary Table 1**). The PCR-amplified fragment was ligated into the pGEM-T easy vector (Promega) and transformed into competent *Escherichia coli* TOP10 cells. Recombinant plasmid DNA was sequenced and compared with GenBank sequences using BLAST (http://www.ncbi.nlm.nih.gov/BLAST/).

The molecular mass and theoretical isoelectric point were predicted using the ProtParam tool at the ExPASy server (http://web.expasy.org/protparam/). Signal peptides were predicted using SignalP (http://www.cbs.dtu.dk/services/SignalP/). Transmembrane motifs were determined using TMHMM (http://www.cbs.dtu.dk/services/TMHMM-2.0/), and protein motifs were predicted using Motif Scan (http://hits.isb-sib.ch/cgi-bin/motif_scan) (Zhai et al., 2016).

### Expression and Identification of r*Et*eIF-5A Protein

The PCR fragment was ligated into the prokaryotic expression vector pGEX-4T-1 digested with the same restriction endonucleases (*EcoR*I and *Xho*I), to construct the recombinant expression plasmid pGEX-4T-*Et*eIF-5A after sequence. The recombinant plasmid pGEX-4T-*Et*eIF-5A was transformed into *E. coli* BL21 (DE3), and protein expression was induced with 1 mM isopropyl-thio-α-D-galactoside (Sigma-Aldrich, St. Louis, USA) for 8 h at 37°C. The bacterial suspension was centrifuged for 10 min at 10,000 rpm (Ren et al., 2010) at 4°C, and the pellet was collected and resuspended in 40 ml PBS. The bacterial cells were lysed by sonication for 40 min. The bacterial suspension was centrifuged for 10 min at 10,000 rpm at 4°C. The supernatant and pellet were sonicated and analyzed by SDS-PAGE. The soluble r*Et*eIF-5A was purified using GST resin (Beyotime), and protein concentration was estimated using the BCA Protein Assay Kit (Beyotime).

Western blotting was used to detect whether the purified r*Et*eIF-5A has good antigenicity. Proteins were separated by SDS-PAGE and transferred electrophoretically to polyvinylidene difluoride (PVDF) membranes (Millipore, Darmstadt, Germany). Membranes were blocked with 5% skimmed milk in PBS for 2 h at 37°C and incubated with anti-sporozoite rabbit serum (1:100) previously obtained in our laboratory (Han et al., 2015) and GST monoclonal antibody (1:1,000) (Beyotime) (Zhai et al., 2016) for 2 h at 37°C. After that, membranes were incubated with horseradish peroxidase (HRP)-conjugated AffiniPure goat anti-rabbit IgG (H+L) and HRP-conjugated Affinipure goat anti-mouse IgG (H+L) (Proteintech, USA, 1:5,000) for 1 h at 37°C. Immunoreactive bands were visualized by UV transillumination using the ChemiDoc Touch imaging system (Bio-Rad Laboratories, California, USA).

### Production of Anti-r*Et*eIF-5A Serum

Two hundred micrograms of purified r*Et*eIF-5A emulsified in Freund’s complete adjuvant (Sigma-Aldrich) were injected into two 2-month-old New Zealand white male rabbits and 50 μg were injected into seven 6-week-old male mice. After 2 weeks, rabbits and mice were injected with the same dose of purified r*Et*eIF-5A emulsified in Freund’s incomplete adjuvant (Sigma-Aldrich), followed by two booster injections weekly. Blood was collected from carotid artery of two immunized rabbits after general anesthesia. Then we incubated the blood at 37°C for 2 h, maintained at 4°C for 4 h, centrifuged at 3,500 rpm for 12 min, the supernatant was the polyclonal antibody we need.

### Transcript and Protein Level of *Et*eIF-5A at Different Developmental Stages of *E. tenella*

The transcriptional expression of *Et*eIF-5A at different developmental stages of *E. tenella* (UO, SO, Spz, and Mrz) was evaluated by quantitative real-time PCR (qPCR). qPCR amplification was performed on an Eppendorf Mastercycler ep Realplex thermal cycler (Eppendorf, Hamburg, Germany). 18S rRNA and *Et*eIF-5A primers were designed using the generative quantitative PCR design tool by Sangon. Total RNAs were extracted from four developmental stages of *E. tenella* with TRIzol Reagent (Invitrogen) and treated with DNase I (Invitrogen). Then the first-strand cDNA samples were synthesized from DNaseI-treated total RNAs using SuperScript™ II Reverse Transcriptase (Invitrogen) and random pd(N)6 primer. The first strand of 20 ng cDNA was used as the template, and the reagents were added and mixed according to the TaKaRa fluorescence quantitative PCR kit to detect the transcriptional level of *Et*eIF-5A in the four stages of *E. tenella*. The experiment was performed in the dark. All experiments were performed in triplicate and repeated independently three times. Expression levels were normalized using 18S rRNA (Thellin et al., 1999; Walker et al., 2015). The relative expression levels of *Et*eIF-5A were measured using the 2^-ΔΔCt^ method (Michaelidou et al., 2013).

Western blotting was performed to analyze the protein level of *Et*eIF-5A. Whole proteins were extracted from 100mg four different developmental stages of *E. tenella* (UO, SO, Spz, and Mrz) with 300 μl RIPA buffer (Beyotime) and 3 μl protease inhibitor cocktail (Sigma). Protein concentration was determined using the BCA Protein Assay Kit (Beyotime).

All the proteins were then resolved with 12% SDS-PAGE and detected with western blotting with rabbit anti-r*Et*eIF-5A polyclonal antibody (1:100 dilution) and mouse monoclonal anti-α-tubulin antibody (1:1000) (Sigma-Aldrich) and using corresponding second antibodies, as described above. Expression levels were normalized with α-tubulin (Péroval et al., 2006). Image J and GraphPad Prism version 6.0 were used to process the results by western blot in gray analyze.

### Protein Localization and Co-Localization by Indirect Immunofluorescence

#### Localization

To determine the subcellular localization of *Et*eIF-5A in sporozoites and second-generation merozoites, these parasitic forms were transferred to glass slides and air-dried, as previously described (Peroval et al., 2006; Jiang et al., 2012a). DF-1 cells were infected with freshly excysted sporozoites to analyze the distribution of *Et*eIF-5A at different developmental stages. At 2, 24, 48, 60, and 72 h p.i., DF-1 cells were collected, washed, transferred to glass slides, and air-dried. The slides were fixed in 2% paraformaldehyde for 30 min, permeabilized with 1% Triton^®^ X-100 for 25 min, and blocked with 2% BSA in PBS for 2 h at 4°C. After that, the slides were incubated with rabbit anti-r*Et*eIF-5A polyclonal antibody (1:100) for 2 h at 37°C and with Alexa Fluor™ 488 chicken anti-rabbit IgG (H+L) (Invitrogen) (1:500) for 1 h at 37°C in the dark. Nuclei were stained with 4,6-diamidino-2-phenylindole (Beyotime) (15 μg/ml) for 10 min at room temperature. After each step, slides were washed six times with PBS containing 0.5% (v/v) Tween-20 and mounted using 100 μl fluoromount aqueous mounting medium (Sigma-Aldrich). Fluorescence was quenched with 1,4-diazabicyclo [2.2.2] octane (Sigma), and the slides were observed under a fluorescence microscope (model LSM800, Carl Zeiss, Germany). As a control, the co-localization of *Et*eIF-5A and *E. tenella* Ubiquitin (*Et*Ub) which located on the surface of sporozoites at 48 h p.i., mouse anti-r*Et*Ub polyclonal antibody was obtained in our laboratory (Li Z. H. et al., 2020). Rabbit anti-r*Et*eIF-5A polyclonal antibody was replaced by healthy rabbit IgG rabbit as the negative control.

#### Co-Localization

For co-localization experiments, sporozoites, second-generation merozoites, and slides containing DF-1 cells infected with sporozoites for 2 h, 24 h were used. Cells were fixed in 2% paraformaldehyde, permeabilized in TritonX-100, blocked in 5% skimmed milk in PBS, incubated with mouse anti-r*Et*eIF-5A polyclonal antibody (1:100) and rabbit anti-r*Et*CDPK4 polyclonal antibody (1:100), then labeled with Alexa Fluor™ 647 chicken anti-mouse IgG (H+L) (1:200) and Alexa Fluor™ 488 chicken anti-rabbit IgG (H+L) (1:200 ratio dilution) (Invitrogen). A negative control of healthy rabbit IgG was used instead of the mouse anti-r*Et*eIF-5A polyclonal antibody (1:100) and rabbit anti-r*Et*CDPK4 polyclonal antibody (1:100). After each step, slides were washed six times with PBS containing 0.5% (v/v) Tween-20. Nuclei staining and fluorescence quenching were performed as above.

### Analysis of Protein Secretion

Freshly excysted sporozoites (4×10^6^) were resuspended in 100 μl of complete medium (CM), cultured in this medium for 2 h at 41°C, and treated with 5, 10, and 20 μM of staurosporine or DMSO, as described previously (Carruthers et al., 1999). Sporozoites were pelleted by centrifugation for 10 min at 2,800 rpm, and the supernatant was collected. The supernatants were performed and analyzed with western blotting using a rabbit anti-r*Et*eIF-5A antibody and rabbit anti-r*Et*MIC2 antibody generated previously in our laboratory. Rabbit serum raised against the micronemal protein *Et*MIC2 was used as the control (Yan et al., 2018).

### Invasion Inhibition Assay *In Vitro*

This assay was performed to assess the inhibition of the invasion of DF-1 cells by sporozoites, as previously described (Jiang et al., 2012b; Wilson et al., 2015). Rabbit anti-r*Et*eIF-5A polyclonal antibodies were purified with protein A+G beads (Beyotime), and protein concentration was determined using the BCA Protein Assay Kit (Beyotime). Freshly excysted sporozoites were labeled using the Vybrant™ CFDA SE Cell Tracer Kit (Thermo Scientific™) according to the manufacturer’s instructions and resuspended in DMEM containing 5% FBS and 500 U/ml penicillin/streptomycin. Labeled sporozoites were resuspended in 1 ml CM (Gibco, Grand Island, NY, USA). Sporozoites treated with pre-immune rabbit IgG were used as the control, and sporozoites incubated with no antibody were the positive control. 6.0×10^5^ sporozoites were incubated with rabbit anti-r*Et*eIF-5A polyclonal antibody at a concentration of 50, 100, 200, 300, or 400 μg/ml for 2 h, and 2.0×10^5^ cells DF-1 cells were infected for 16 h. Cell invasion was monitored by flow cytometry (Beckman Coulter, USA). Uninfected cells served as the control. Infected cells, uninfected cells, and free sporozoites were gated using RXP software (Beckman Coulter, USA) to count infected (labeled sporozoites) and uninfected (fluorescence-free) cells. The inhibition rate was determined according to the formula: inhibition = 100% × [1 – (% infected cells^antibody treatment^/%infected cells^negative control^)] (Jahn et al., 2009).

### Statistical Analysis

Statistical analysis was performed using SPSS version 22 for Windows (SPSS, Chicago, Illinois, USA), Microsoft Office Excel version 2013 for Windows (Redmond, Washington, USA), and GraphPad Prism version 6.0 (GraphPad, La Jolla, CA, United States). The relative expression of the target gene was determined using the 2^–ΔΔCt^ method. Differences between the study groups were analyzed using one-way analysis of variance and Duncan’s multiple range test. Two-tailed p-values of less than 0.05 were considered statistically significant.

## Results

### Screening of Proteins Interacting With *Et*CDPK4 by Co-IP, His Pull-Down, and Mass Spectrometry

**Figure 1:**
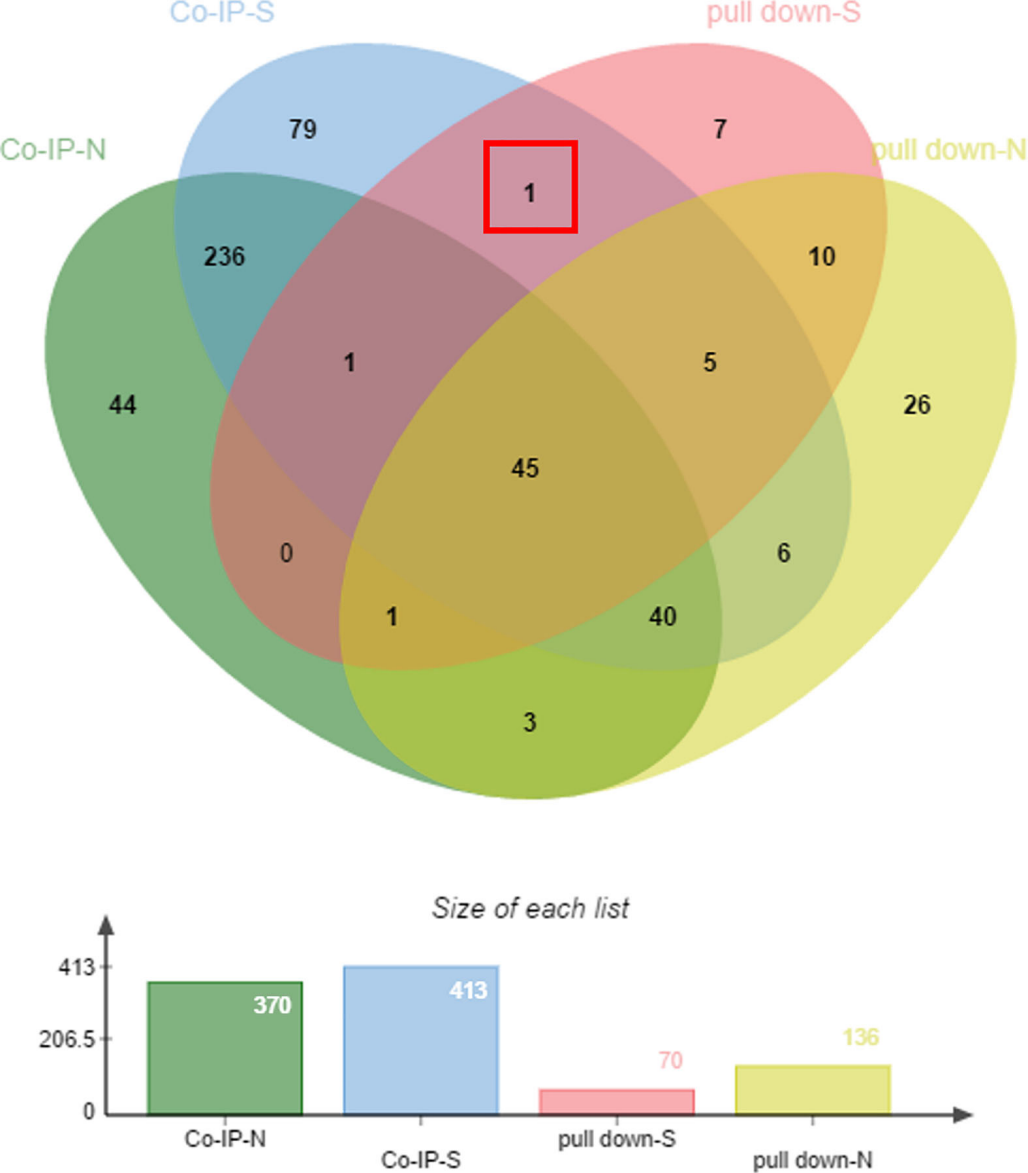
Mass spectrometry analysis of co-immunoprecipitated and pulled-down fractions. pull-down-S, positive control of pull down; pull-down-N, negative control of pull down; co-IP-S, positive control of co-IP; co-IP-N, negative control of co-IP.

The samples enriched in co-IP and His pull-down assays were separated by SDS-PAGE. The gels were silver stained (**Supplementary Figure 1**), silver stained results showed that there were no distinct bands of specificity, therefore the whole strip was removed for mass spectrometry and analyzed. The proteins were subject to blast analysis by shotgun, then compared using the UniProt database (Huang et al., 2018). Peptides were analyzed against a UniProt protein database, and the results are shown in **Supplementary Data 1**. In co-IP and His pull-down assays, 70 proteins were found in the sample group of the His pull down assay, 136 proteins from the negative His pull down assay, 413 proteins bound to anti-r*Et*CDPK4 IgG of co-IP assay, and 370 proteins bound to pre-immune rabbit IgG of co-IP assay. We collated the analysis results using a Venn diagram and found that the protein, translation initiation factor eIF-5A (ETH_00015190), showed positive interaction with *Et*CDPK4 by both co-IP and the pull down assays (**Figure 1**, red box).

### Characterization of *Et*eIF-5A

The ORF of the *Et*eIF-5A gene was amplified by PCR using cDNA from SO. Bioinformatics analysis showed that the cDNA sequence of *Et*eIF-5A was 486 base pairs and encoded 161 amino acids. The predicted molecular weight was 17.3 kDa, and the isoelectric point was 5.35. The protein has no signal peptide and transmembrane domains. The protein contains an amidation site (residues 67-70), a casein kinase II phosphorylation site (residues 2-5), two N-myristoylation sites (residues 15-20 and 141-446), three protein kinase C phosphorylation sites (residues 47-49, 50-52, and 67-69), one translation initiation factor 5A (residues 18-158), one 8-hydroxy 2, 7, 10-triaminodecanoic acid (hypusine) signal region (residues 50-57) of eukaryotic start factor 5A, one bacterial Ig like domain 1 (residues 1-10), one KOW motif (residues 29-61), one eukaryotic start factor 5A hypusine, and one oligonucleotide/oligosaccharide-binding fold motif (residues 85-159) (**Supplementary Figure 2**).

The amino acid sequence was homologous to translation initiation factor eIF-5A from *E. tenella* (XP_013229451.1) (100%), *E. brunetti* (CDJ47139.1) (93%, 149/161), *E. maxima* (XP_013335634.1) (91%, 147/161), and *E. acervulina* (XP_013249451.1) (91%, 146/161), and presented 75% homology with the translation initiation factor eIF-5A from *Plasmodium malariae* (Sequence ID: XP_028863531.1), *Plasmodium reichenowi* (Sequence ID: XP_012763921.1), and *Plasmodium gaboni* (Sequence ID: XP_018640500.1).

### Expression and Identification of r*Et*eIF-5A Protein

**Figure 2:**
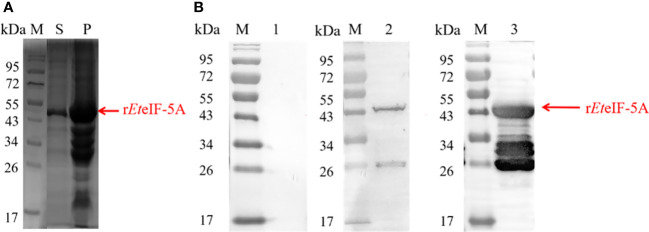
Antigenicity of r*Et*eIF-5A. **(A)** SDS-PAGE stained of purified r*Et*eIF-5A by coomassie bright blue. **(B)** Western blot analysis of purified r*Et*eIF-5A. Rabbit antiserum against *E. tenella* sporozoites or monoclonal anti-glutathione S-transferase (GST) antibody was used as the primary antibody. Lane 1, r*Et*eIF-5A probed with pre-immune rabbit serum. Lane 2, r*Et*eIF-5A probed with anti-sporozoite rabbit serum. Lane 3, r*Et*eIF-5A probed with monoclonal anti-GST antibody. S, supernatant; P, precipitation.

Recombinant protein r*Et*eIF-5A was expressed in *E. coli* BL21(DE3) in soluble form and precipitate (**Figure 2A**). Western blot using anti-sporozoite rabbit serum and anti-GST monoclonal antibody showed one 43 kDa protein and smaller proteins, it is possible that the protein was degraded during purification. None of the recombinant proteins reacted with pre-immune rabbit IgG. These results indicate that r*Et*eIF-5A has good antigenicity and is degraded (**Figure 2B**).

### Validation

#### Validation of the Results of BiFC Assays

**Figure 3:**
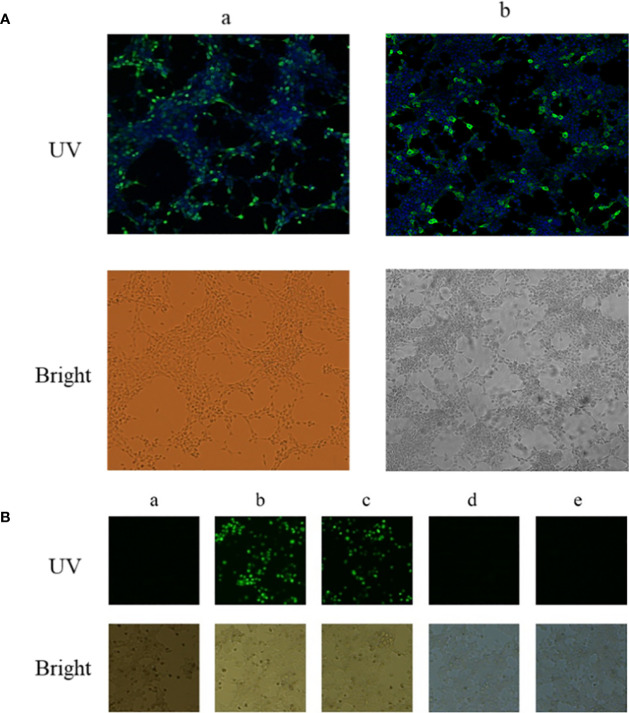
Interaction between *Et*CDPK4 and *Et*eIF-5A by bimolecular fluorescence complementation. **(A)** 293T cells were transfected with pBiFC-VN155-*Et*eIF-5A or pBiFC-VC155-*Et*CDPK4 and analyzed with IFA using antisera r*Et*eIF-5A and antisera r*Et*CDPK4: (a) pBiFC-VN155-*Et*eIF-5A transfected into 293T cells; (b) pBiFC-VC155-*Et*CDPK4 transfected into 293T cells. In UV figures, pBiFC-VN155-*Et*eIF-5A or pBiFC-VC155-*Et*CDPK4 showed with green fluorescence, the nucleus stained to blue fluorescence. **(B)** Interaction between *Et*CDPK4 and *Et*eIF-5A by bimolecular fluorescence complementation. a, negative control; b, positive control; c, pBiFC-VC155-*Et*CDPK4 and pBiFC-VN155-*Et*eIF-5A co-transfected into 293T cells; d, pBiFC-VC155-*Et*CDPK4 and pBiFC-VN155 co-transfected into 293T cells; e, pBiFC-VN155-*Et*eIF-5A and pBiFC-VC155 co-transfected into 293T cells.

A BiFC assay was performed to confirm the interaction between *Et*eIF-5A and *Et*CDPK4 in living cells. The recombinant plasmids pBiFC-VN155-*Et*eIF-5A and pBiFC-VC155-*Et*CDPK4 were independently transfected into 293T cells, and protein expression was analyzed by indirect immunofluorescence. The results indicate that the two recombinant proteins were successfully expressed in the 293T cells, respectively (**Figure 3A**).

pBiFC-VN155-*Et*eIF-5A and pBiFC-VC155-*Et*CDPK4 were jointly transfected into 293T cells. Forty-eight hours after transfection, the results were analyzed by fluorescence microscopy. The negative control had no fluorescence, and the positive control had a green fluorescence (**Figure 3B**, panels a and b). 293T cells co-transfected with pBiFC-VC155-*Et*CDPK4 and pBiFC-VN155-*Et*eIF-5A emitted green fluorescence (**Figure 3B**, panel c). 293T cells co-transfected with pBiFC-VC155-*Et*CDPK4 + pBiFC-VN155 or pBiFC-VN155-*Et*eIF-5A + pBiFC-VC155 were used as controls and emitted no fluorescence (**Figure 3B**, panel d and e). The BiFC results showed that *Et*CDPK4 interacted with *Et*eIF-5A.

#### Validation of the Results of GST Pull-Down Assays

A GST pull-down assay was performed to confirm the interaction between *Et*eIF-5A and *Et*CDPK4 *in vitro*, and the results were analyzed by western blotting. Two bands were detected in the blots probed with rabbit anti-r*Et*eIF-5A and anti-r*Et*CDPK4 serum (**Figure 4.2**), corresponding to r*Et*eIF-5A (lower band) and r*Et*CDPK4 (upper band) (**Figures 4.3** and **4.4**), indicating that r*Et*eIF-5A was bound to r*Et*CDPK4. The negative control selected the GST protein with r*Et*CDPK4, and but no band was detected (**Figure 4.1**); Blank control only using PBS buffer with r*Et*eIF-5A, and western blot detected no bands appeared (**Figure 4.5**). The GST pull down results showed that *Et*CDPK4 interacted with *Et*eIF-5A.

**Figure 4:**
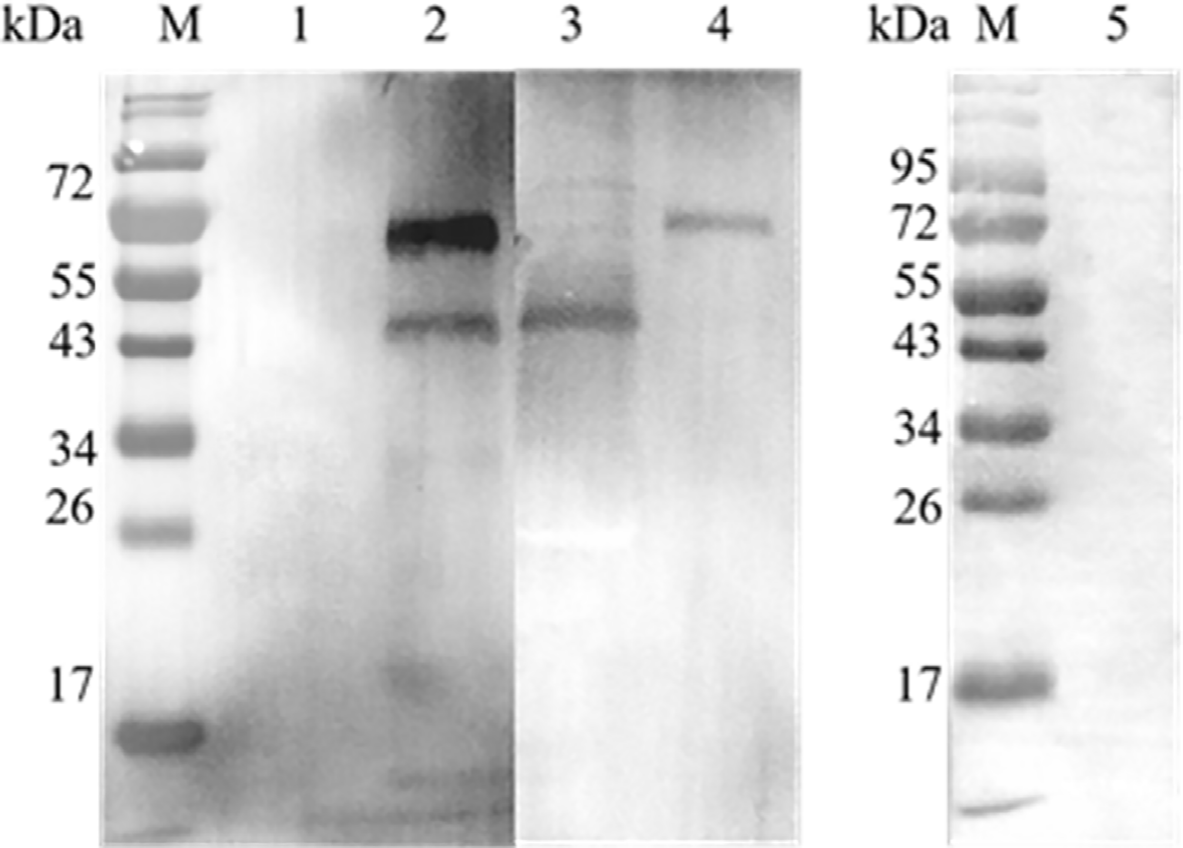
Analyze the results of GST pull down by Western blot. M, Molecular weight marker. Lane 1, glutathione S-transferase (GST)-tag incubated with r*Et*CDPK4, detected with anti-r*Et*eIF-5A and anti-r*Et*CDPK4 rabbit serum as a negative control; Lane 2, r*Et*CDPK4 incubated with r*Et*eIF-5A, detected with anti-r*Et*eIF-5A and anti-r*Et*CDPK4 rabbit serum as the sample group; Lane 3, purified protein of r*Et*eIF-5A probed with anti-r*Et*eIF-5A and anti-r*Et*CDPK4 rabbit serum; Lane 4, purified protein of r*Et*CDPK4 probed with anti-r*Et*eIF-5A and anti-r*Et*CDPK4 rabbit serum; Lane 5, PBS with r*Et*eIF-5A loaded in an empty glutathione-agarose column, detected with anti-r*Et*CDPK4 rabbit serum and anti-r*Et*eIF-5A rabbit serum as a blank control.

#### Validation of the Results of Co-IP Assays

A co-IP assay was performed to confirm the interaction between *Et*eIF-5A and *Et*CDPK4 *in vitro*. Western blotting showed two bands corresponding to r*Et*eIF-5A (lower band) and r*Et*CDPK4 (upper band) (**Figure 5.2**), which are the same molecular weight as r*Et*eIF-5A (**Figure 5.3**) and r*Et*CDPK4 (**Figure 5.4**). For the negative control, we did not change the sample but used the negative resin provided in the kit. No bands were detected in the negative control (**Figure 5.1**). No bands were detected in the blank control (pre-immune rabbit IgG) (**Figure 5.5**).

**Figure 5:**
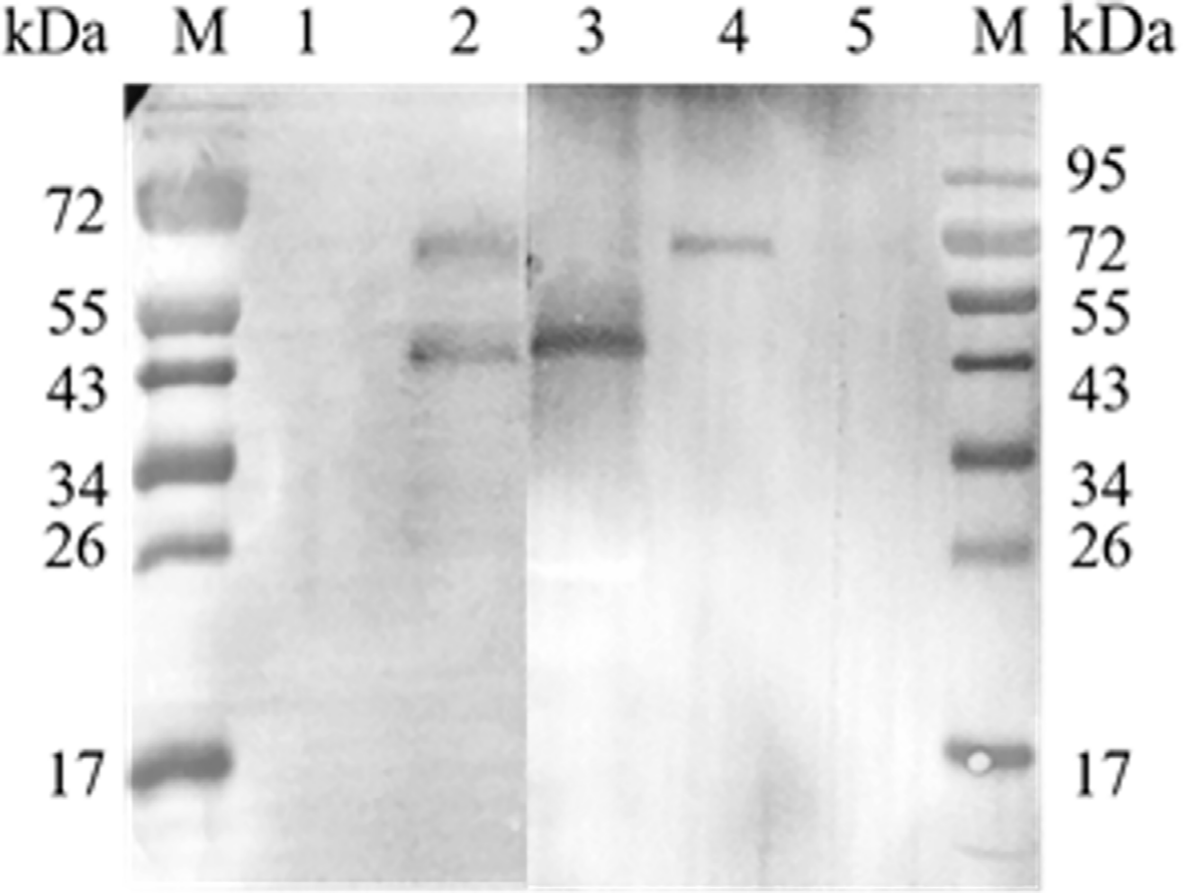
Co-immunoprecipitation of *Et*CDPK4 and *Et*eIF-5A. M, molecular weight marker; 1, negative control; 2, blot probed with anti-r*Et*eIF-5A and anti-r*Et*CDPK4 serum (Sample group); 3, purified protein of r*Et*eIF-5A; 4, purified protein of r*Et*CDPK4; 5, blank control with pre-immune rabbit IgG.

### Co-Localization

**Figure 6:**
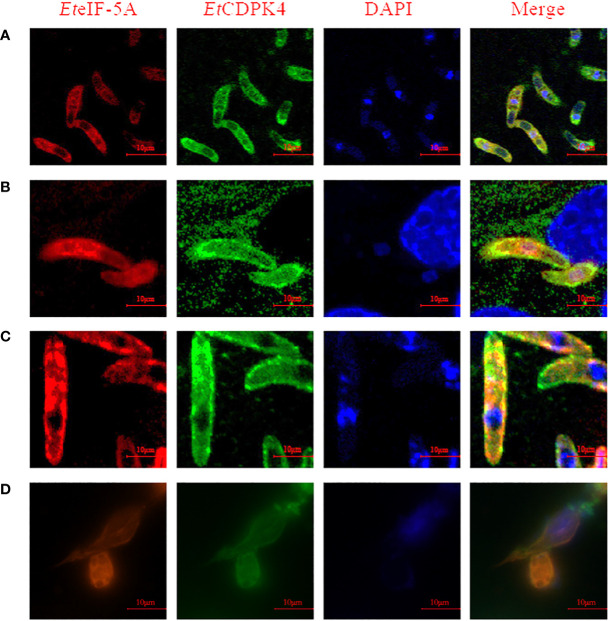
Co-localization of *Et*eIF-5A and *Et*CDPK4 in *Eimeria tenella*. **(A)** Sporozoites before cell invasion; **(B)** Sporozoites at 2 h post infection; **(C)** Second-generation merozoites; **(D)** Trophozoites at 2 h post infection.

A co-localization assay was performed to localize *Et*eIF-5A and *Et*CDPK4. Rabbit anti-r*Et*CDPK4 and mouse anti-r*Et*eIF-5A polyclonal antibody were used as primary antibodies. Alexa Fluor 647-labeled chicken anti-mouse IgG and Alexa Fluor 488-labeled chicken anti-rabbit IgG were used as secondary antibodies to localize *Et*eIF-5A (red fluorescence) and *Et*CDPK4 (green fluorescence), respectively. The co-localization of *Et*CDPK4 and *Et*eIF-5A is shown in **Figure 6**. In sporozoites before invasion DF-1 cells, *Et*eIF-5A and *Et*CDPK4 were evenly distributed throughout the cytoplasm, but not in the refractile body. After sporozoites invasion, DF-1 cells 2 h, *Et*eIF-5A was mainly located in the cytoplasm of parasites except for the refractile body, while *Et*CDPK4 was mainly distributed on the surface and apical end of sporozoites and the fluorescence increased (**Figures 6A, B**, respectively). In second-generation merozoites (**Figure 6C**), *Et*eIF-5A was mainly located in the cytoplasm, whereas *Et*CDPK4 was located in the cytoplasm and on the surface. After sporozoites developed at 24 h post invasion, *Et*CDPK4 and *Et*eIF-5A was also mainly located in the cytoplasm of trophozoite (**Figure 6D**). *Et*CDPK4 and *Et*eIF-5A function in the same location and may work together during the different developmental stages of *E. tenella* and when the sporozoites developed in the cell.

### Transcript and Protein Level of *Et*eIF-5A

**Figure 7:**
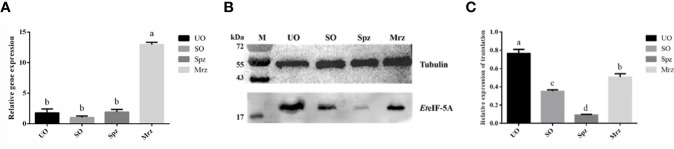
Transcript and protein level of *Et*eIF-5A. UO, unsporulated oocysts; SO, sporulated oocysts; Spz, sporozoites; Mrz, second-generation merozoites. Anti-α-tubulin antibody was used as a loading control. The lysate of different stages of *E. tenella* was probed with rabbit anti-r*Et*eIF-5A. mRNA expression levels **(A)** and protein expression levels **(B, C)** of *Et*eIF-5A at four developmental stages of *E. tenella*. Data are means and standard deviations of three independent experiments performed in triplicate.

The transcription level of *Et*eIF-5A mRNA was analyzed by qPCR. *Et*eIF-5A was transcribed and expressed in four developmental stages of *E. tenella*. The transcription level of *Et*eIF-5A was significantly higher in second-generation merozoites (**Figure 7A**). α-tubulin was used as the housekeeping gene. The protein level of *Et*eIF-5A was the highest in UO and lowest in sporozoites (**Figures 7B, C**).

### Localization of *Et*eIF-5A by Immunofluorescence

**Figure 8:**
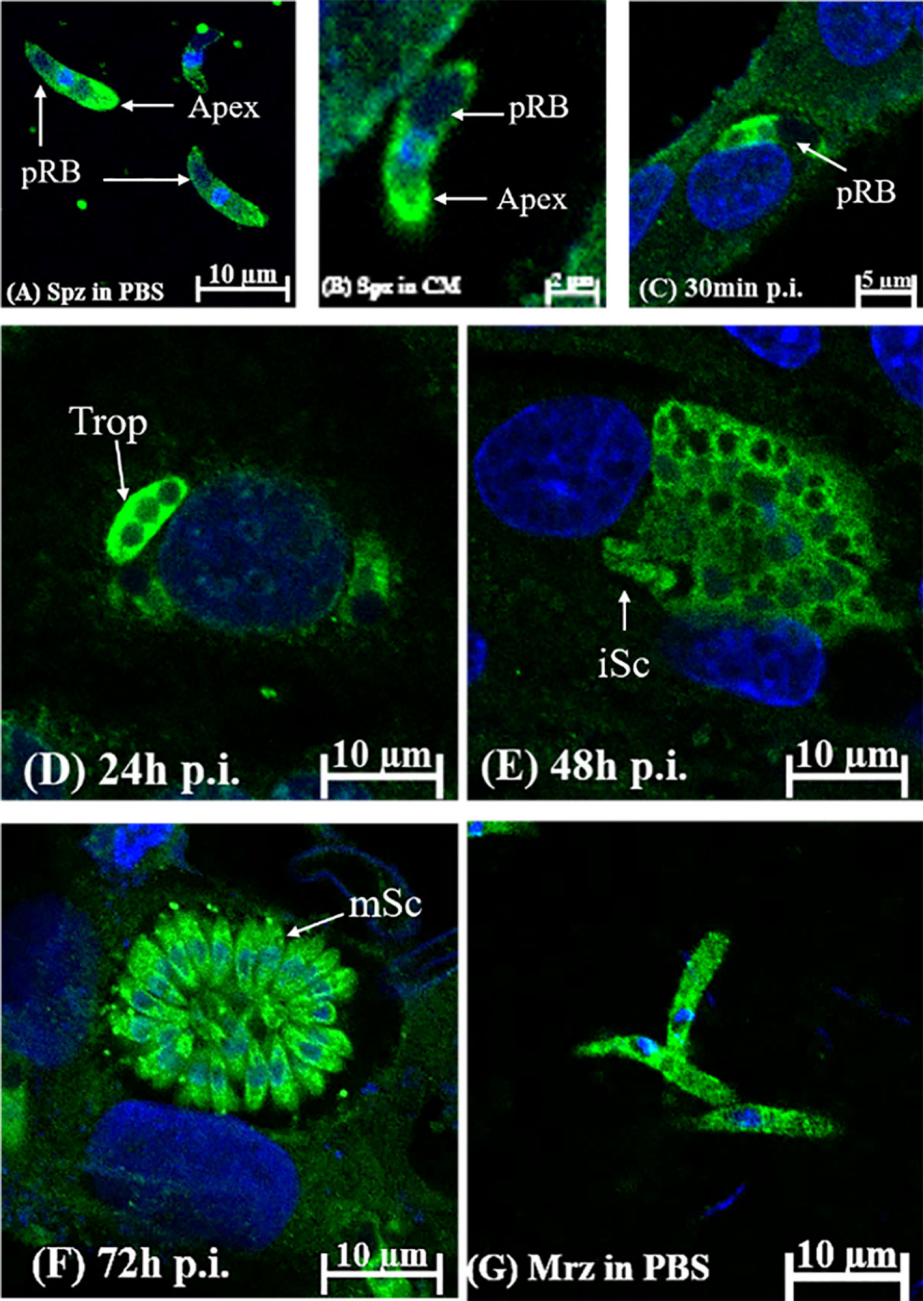
Localization of *Et*eIF-5A in different developmental stages of *Eimeria tenella* by indirect immunofluorescence using rabbit anti-r*Et*eIF-5A IgG. **(A)** sporozoites (Spz) in phosphate-buffered saline (PBS); pRB, posterior refractile body. **(B)** sporozoites in complete medium. DF-1 cells were collected at the indicated time points post-infection (p.i.). **(C)** sporozoites at 30 min p.i.; **(D)** trophozoite (trop) at 24 h p.i.; **(E)** immature schizont (iSC) at 48 h p.i.; **(F)** mature schizont (mSc) at 72 h p.i.; **(G)** merozoites (Mrz) in PBS.

The localization and distribution of *Et*eIF-5A were determined *in vitro* by immunofluorescence. *Et*eIF-5A was localized at the within the cytoplasm but not in the refractile body (**Figure 8A**) and throughout the cytoplasm of the second-generation merozoites (**Figure 8G**). The fluorescence signal was distributed evenly in sporozoites incubated in complete medium for 2 h (**Figure 8B**). *Et*eIF-5A was mainly localized in the cytoplasm of sporozoites except for refractile bodies at 30 min pi (Liu et al., 2019) (**Figure 8C**). At 24 h pi, *Et*eIF-5A was localized primarily to the cytoplasm of trophozoites, and fluorescence was stronger than at 30 min pi (**Figure 8D**). At 48 h pi, *Et*eIF-5A was mainly localized in the cytoplasm of immature schizonts, and fluorescence was weaker (**Figure 8E** and **Supplementary Figure 3**). At 72 h pi, *Et*eIF-5A was mainly localized in the cytoplasm of mature schizonts (Li C. et al., 2020), and fluorescence was stronger than at 48 h pi (**Figure 8F**). No obvious fluorescence was seen in the negative control group (**Supplementary Figure 4**).

### Secretion of *Et*eIF-5A

**Figure 9:**
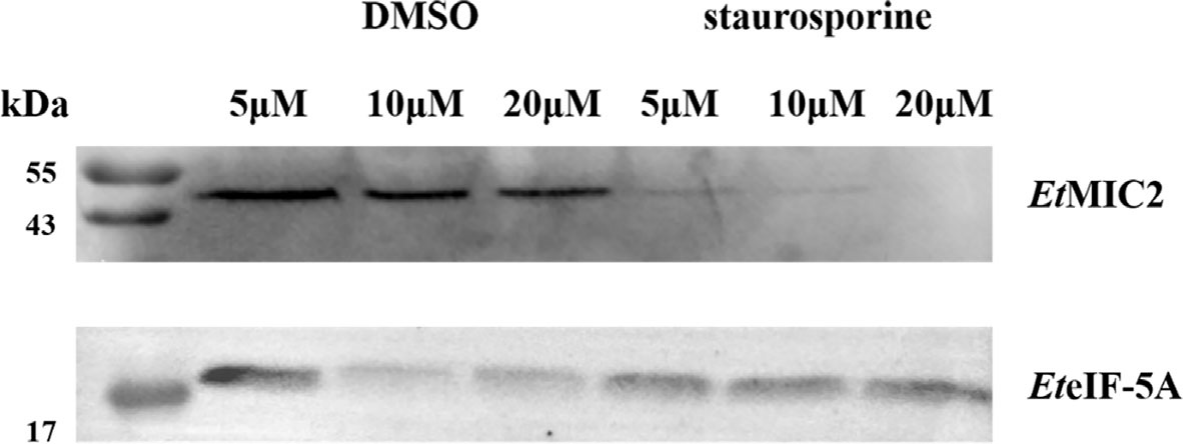
Western blot of secretion experiment. Upper panel: rabbit serum raised against the micronemal protein *Et*MIC2 (control). Lower panel: rabbit serum raised against the protein *Et*eIF-5A.

Based on the localization of *Et*eIF-5A in parasite host cells, we speculate that *Et*eIF-5A may be a secreted protein, and a secretion assay was performed to confirm this hypothesis. Freshly excysted sporozoites were incubated in medium supplemented with 5, 10, and 20 μM DMSO, and 5, 10, and 20 μM staurosporine for 2 h, centrifuged, and analyzed by western blotting with rabbit anti-r*Et*eIF-5A serum. Rabbit anti-r*Et*MIC2 serum served as the control. *Et*eIF-5A expression was not affected by different concentrations of staurosporine, indicating that this protein is a secreted protein but not from micronemes (**Figure 9**).

### Inhibition of Host Cell Invasion Using r*Et*eIF-5A Polyclonal Antibodies

**Figure 10:**
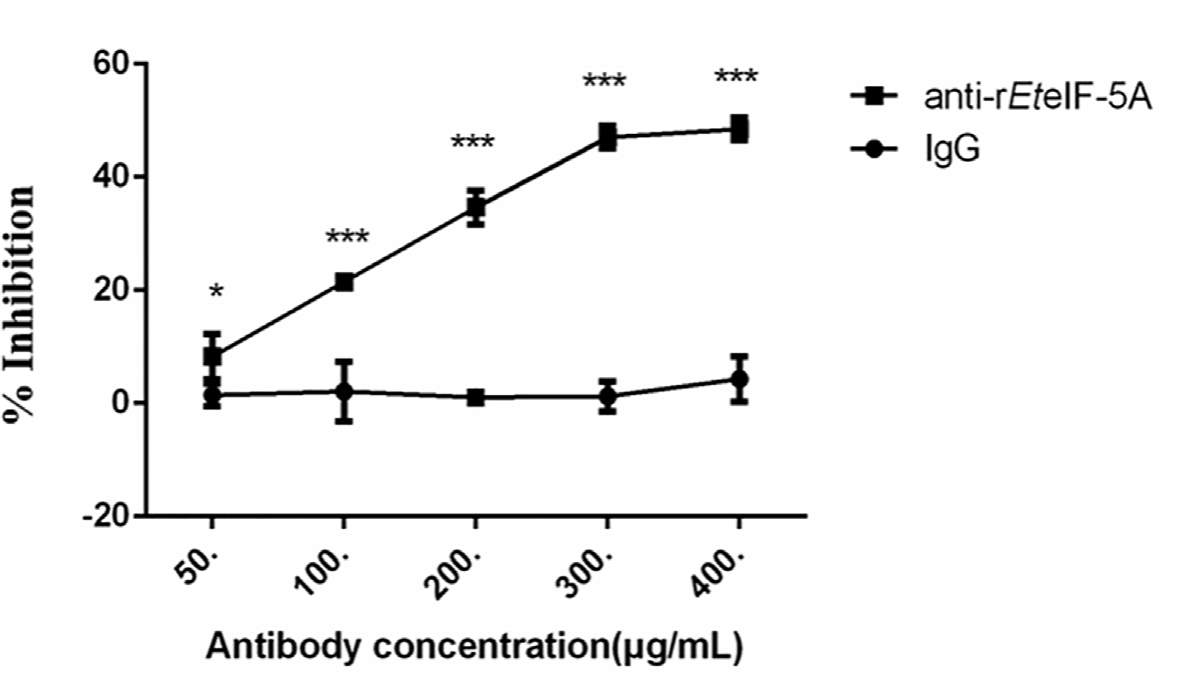
Inhibition of host cell invasion *in vitro* using anti-r*Et*eIF-5A antibody. Anti-r*Et*eIF-5A, rabbit anti-r*Et*eIF-5A IgG antibody; IgG, pre-immune rabbit serum. All assays were performed in triplicate. In figures, significant differences (p < 0.01) between groups are indicated by different numbers of ‘*’. Highly significant differences (p < 0.001) between groups are indicated by different numbers of ‘***’.

*In vitro* inhibition experiments were performed to assess the effect of *Et*eIF-5A on sporozoite invasion of DF-1 cells. Compared with pre-immune rabbit IgG, the inhibition rate increased significantly as rabbit anti-r*Et*eIF-5A IgG concentration increased (**Figure 10**). The inhibition rate reached approximately 48% at the IgG concentration of 400 μg/ml, demonstrating that *Et*eIF-5A might play an important role in host cell invasion by sporozoites.

## Discussion

Protein complexes exert multiple biological functions, including gene expression regulation, signal transduction, metabolism, cell proliferation, and apoptosis (Zhang, 2009). Studies have shown that the study of protein interactions can help elucidate biological processes, pathological mechanisms, and so on (Man-Sheng et al., 2010). Many methods are currently used for screening interacting proteins, including the yeast two-hybrid (Y2H) system, co-immunoprecipitation, and GST pull-down. In addition, co-immunoprecipitation followed by mass spectrometry is a fast, sensitive, and reliable method to identify protein interactors and discover new interaction partners undetectable by Y2H analysis (Free et al., 2009). Putative interactors to *Et*CDPK4 were screened in this study by co-IP and His pull-down followed by mass spectrometry. 70 His-tagged and 136 untagged proteins from the pull-down assay and 413 proteins bound to anti-r*Et*CDPK4 IgG and 370 proteins bound to pre-immune rabbit IgG in co-IP assays were identified. The only protein identified in both assays was *Et*eIF-5A.

Both methods have limitations. Co-IP identifies several types of molecular interactions, including protein-protein interactions. Moreover, the sensitivity of this method is low. Therefore, this technique may not detect weak and transient interactions. For pull-down assays, proteins expressed using prokaryotic expression systems may be folded incorrectly. Furthermore, charge interactions leading to adsorption may influence the structure of fusion proteins and lead to false positives. In addition, protein interactions are complex, and competitive binding between proteins may lead to false negative results (Wang, 2015).

Y2H technology was previously used in our laboratory to screen putative interactors to *Et*CDPK4 (Lv et al., 2018). The analysis identified eight putative interactors, but not *Et*eIF-5A. A previous study showed that Y2H could simulate protein-protein interactions in eukaryotic systems. This technology is simple, sensitive, and widely used for assessing unknown protein-protein interactions (Young, 1998). Nonetheless, this method has some limitations. Y2H analysis protein interactions require localization of proteins in the nucleus so it is not suitable for membrane-associated proteins, integral membrane proteins, many large soluble cytosolic proteins, and proteins localized to other subcellular compartments (Bruckner et al., 2009). While IFA showed that *Et*eIF-5A was mainly localized in the cytoplasm of sporozoites. In addition, other events also can cause the loss of potential interactors by using Y2H. For example, the relatively low mRNAs levels of putative interacting proteins and the defects of library construction (Ferro et al., 2014; Wagemans and Lavigne, 2015). So further efforts are needed to study the interaction mechanism of *Et*eIF-5A and *Et*CDPK4.

The interaction between *Et*eIF-5A and *Et*CDPK4 was validated using BiFC, co-IP, and GST pull-down. BiFC can localize proteins allows the rapid visualization of subcellular localization of proteins (Shi et al., 2013). The interaction between *Et*eIF-5A and *Et*CDPK4 in living cells was confirmed by BiFC. In addition, Co-IP and GST pull-down increased the reliability of the experimental results and reduced experimental errors. Co-localization was used to show the interaction *in vivo* preliminarily. In the results, we found that *Et*eIF-5A and *Et*CDPK4 are mainly located in the cytoplasm of sporozoites and trophozoites. After sporozoites DF-1 cells 2 h, *Et*eIF-5A was mainly located in the cytoplasm of parasites, while *Et*CDPK4 was mainly distributed on the surface and apical end of sporozoites besides in the cytoplasm. In addition, the localization of these two proteins was also inconsistent in merozoites. So we supposed that *Et*eIF-5A and *Et*CDPK4 have their own specific functions in addition to working together with interaction in different developmental stages of *E. tenella*. The biological functions of proteins are generally completed by complexes, that is, through the interaction of proteins. But how the interacted proteins exactly effect the invasion is unclear.

eIF-5A is highly conserved throughout evolution. In addition, eIF5A is the only protein known to contain the unusual amino acid residue hystidine (Schnier et al., 1991; Chen and Liu, 1997). This protein was originally purified from ribosomes isolated from reticulocyte lysates and was shown to stimulate the synthesis of methoxypuromycin, suggesting that it plays a role in the formation of the first peptide bond during protein synthesis (Benne and Hershey, 1978). In addition, recent studies have shown that eIF-5A interacts with the 80S ribosome and translation elongation factors eEF1A and eEF2 (Jao and Kuang, 2005). Under specific conditions, eIF-5A may participate in cell cycle regulation because the absence of eIF5A increases the number of yeast cells and leads to cell cycle arrest in G1 phase (Kang et al., 1993). It is documented that eIF-5A is closely related to eukaryotic translation elongation factor eEF2 function during translation elongation (Dias et al., 2012), and eIF5A binds to the E and P sites of the small ribosome subunit and acts as peptidyl transferase, possibly in synergy with eEF2 (Rossi et al., 2016; Schmidt et al., 2016). The loss of eIF5A may also lead to an increase in ribosome transit time, indicating that eIF5A is involved in translation elongation (Zanelli et al., 2006). A previous report showed that eIF5A modifies tyrosine (Barbosa et al., 2016). So, we supposed that *Et*eIF5A can potentially modify *Et*CDPK4, thereby affecting cell invasion by sporozoites.

Amino acid sequence analysis indicated that *Et*eIF5A has a translation initiation factor 5A and a eukaryotic initiation factor 5A hypusine signal region. At the beginning of translation, the signal region is involved in the synthesis of methylpuromycin, translation elongation, and ribosome-stalling sequences, including polyproline (Rossi et al., 2016). A study showed that a myristoylated peptide from β-protein kinase C (PKC) pseudosubstrate selectively inhibited a PKC subtype in human fibroblasts (Eichholtz et al., 1993). The inhibition of myristoylated peptide was moderate but significantly affected the invasion of DF-1 cells by *E. tenella* (Wang et al., 2016). The bioinformatic analysis of *Et*eIF5A showed that the amino acid sequence contained two N-myristoylation sites and a bacterial Ig-like domain, which sits may cause loosen of enzyme structure. We consider this domain may affect the function of *Et*CDPK4, thereby affecting their interaction (Hao, 2017). Moreover, the oligonucleotide/oligosaccharide-binding fold motif may allow the binding of *Et*eIF5A to nucleic acids of *E. tenella*, thereby controlling its physiological characteristics.

Although *Et*eIF-5A was transcribed and expressed in the four developmental stages, the transcription level of *Et*eIF-5A was high in second-generation merozoites, Protein level in unsporulated oocysts and second-generation merozoites than sporulated oocysts and sporozoites. The results of *Et*eIF-5A mRNA transcription was inconsistent with protein levels, which may be the result of post-transcriptional modification, because eIF5A contains post-translational modifications, which are essential to restore polyproline-mediated ribosome arrest and may initiate translation (Gutierrez et al., 2013). *E. tenella* has a complex life cycle including different developmental stages. A high number of transcripts does not necessarily indicate corresponding amounts of translated protein, which is related to gene functions in the various the parasite stages (Etzold et al., 2014). Western blotting showed that the expression of *Et*eIF-5A was high in unsporulated oocysts and second-generation merozoites. These two stages were developed in the intestine of chickens and need high metabolism to obtain nutrients from the host cells and evade the host’s immune response (Fetterer et al., 2007). So we propose that the high expression of *Et*eIF-5A in the developmental stages in host cells may initiate more proteins translation and synthesis. In a previous report, *Et*CDPK4 was expressed in all four developmental stages of *E. tenella* by using western blot and highly expressed in second-generation merizoites (Wang et al., 2016). So it was supposed that *Et*eIF-5A may affect *Et*CDPK4 through signaling pathways in all developmental stages of *E. tenella*, especially in the second-generation merozoites.

*Et*eIF-5A was mainly localized to the cytoplasm of the parasites. As the sporozoites begin to develop in host cells, the fluorescence intensity is increased, and then gradually decreases. To the stage of releasing the second generation merozoites, the fluorescence intensity increases. The translation and localization results showed that *Et*eIF-5A was highly expressed in late stages of parasite development, which may be because translation initiation factor 5A can accelerate the rate of peptidyl-tRNA hydrolysis by eRF1, which plays a role in the termination of eukaryotic protein translation (Saini et al., 2009; Schuller et al., 2017), therefore leads to differences in the level of transcript and protein.

The secretion experiment proved that *Et*eIF-5A was a secreted protein, but Signal P predicted that it did not contain a signal peptide. In a previous report, Peroval et al. (2006) found that *E. tenella* heat shock protein 90 (*Et*HSP90), which is essential for the invasion of the host cell and schizont growth, was a secreted protein by sporozoites and detected in parasitophorous vacuole membrane (PV). However, the protein had no signal peptide nor a transmembrane region and was also detected in the protein complex. Initial interaction with the cell generates a cascade of events leading to parasite penetration. *Et*Hsp90 may play a role in the cascade of signal transmission permitting it to bring together and maintain secreted apical proteins as heterocomplexes. Interestingly, *Et*CDPK4 was also found in the PV and involved in parasite penetration (Wang et al., 2016). It should be a secreted protein, however *Et*CDPK4 has no signal peptide. So, we supposed that *Et*eIF-5A and *Et*CDPK4 may be secreted with other proteins during the secretion step necessary to invasion. In peptidomics, some peptides are produced within the secretory pathway, based on the presence of an N-terminal signal peptide sequence on the peptide precursor that drives the translocation of the nascent protein into the lumen of the endoplasmic reticulum. These secretory pathway peptides may play a role in cell-cell signaling as neuropeptides and peptide hormones (Reits et al., 2004). Recent studies, however, have shown that several extracellular proteins, such as FGF-1, FGF-2, IL-1 and galectins found in the extracellular matrix, can be exported without a classical N-terminal signal peptide. Furthermore, nuclear HMGB1 and viral proteins, such as HIV-tat or herpes simplex virus VP22, have been shown to enter the non-classical secretory pathway. Secretion of proteins without an N-terminal signal peptide is currently known as leaderless secretion or the non-conventional/non-classical secretory pathway (Gelman et al., 2013). The specific secretory mechanism of *Et*eIF-5A and *Et*CDPK4 is unclear and needs further study.

The invasion inhibition assay results showed that anti-r*Et*eIF-5A antibody significantly inhibited cell invasion by sporozoites. The invasion inhibition assay works by using anti-r*Et*eIF-5A antibody to neutralize *Et*eIF-5A activity. From the results, the inhibition rate was approximately 48%, which indicates that anti-r*Et*eIF-5A antibody has an effect on inhibiting the cell invasion of sporozoites and shows that *Et*eIF-5A plays a certain role in the invasion process. In addition, the anti-r*Et*CDPK4 antibody also can inhibit sporozoite invasion as well (Wang et al., 2016), so we speculate that *Et*eIF-5A may interact with *Et*CDPK4 in sporozoites and participate in cell invasion. eIF5A has been examined on the proliferation and invasion of HCC cells using a specific inhibitor for eIF5A hypusination and by suppressing eIF5A2 using small interfering RNA (siRNA). The results showed that eIF5A can be a reliable prognostic marker for disease severity of HCC patients and can be used as a target for the development of HCC therapy (Lee et al., 2010). In apicomplexan protozoans, CDPKs have been identified as part of the mechanistic link between Ca^2+^ signaling and differentiation, motility, invasion and escape from the host cell (Billker et al., 2009; Moreno et al., 2011). For example, in *Plasmodium falciparum*, *Pf*CDPK1 can phosphorylate Myosin A tail domain-interacting protein (MTIP) and glideosome-associated protein 45 (GAP45), which are the components of the motor complex that providing a driving force for parasites to invade the host (Green et al., 2008). In summary, we supposed *Et*eIF-5A and *Et*CDPK4 influence the invasion process through their interaction. Nonetheless, the mechanism of interaction between these two proteins remains to be explored.

In conclusion, the results of co-IP, GST pull-down, and BiFC indicate that *Et*eIF-5A interacts with *Et*CDPK4. *Et*eIF-5A was highly expressed in UO. *Et*eIF-5A was mainly localized in the cytoplasm of parasites. Invasion inhibition assay results showed that rabbit anti-r*Et*eIF-5A antibody strong inhibited host cell invasion by sporozoites. These results lay the foundation for further research on the mechanism of action of *Et*CDPK4 and *Et*eIF-5A and their role in the life cycle of *E. tenella*.

## Data Availability Statement

The raw data supporting the conclusions of this article will be made available by the authors, without undue reservation.

## Ethics Statement

The animal study was reviewed and approved by Animal Care and Use Committee of the Shanghai Veterinary Research Institute of the Chinese Academy of Agricultural Sciences.

## Author Contributions

HH, HD, and SL generated the idea and designed the project. SL, HD, QZ, SZ, YY, QW, HW, and SY performed the experiments. SL, HD, and BH analyzed the data. SZ, QZ, HD, BH, YY, QW, HW, and SY contributed to collected parasites. SL, HD, and HH wrote the manuscript. All authors contributed to the article and approved the submitted version.

## Funding

This work was supported by the National Natural Science Foundation of China (No. 31970420, 31672551) and National Sharing Service Platform for Parasite Resources (TDRC-2019-194-30).

## Conflict of Interest

The authors declare that the research was conducted in the absence of any commercial or financial relationships that could be construed as a potential conflict of interest.

## Supplementary Material

Supplementary Figure 1 SDS-PAGE analysis of pulled-down and co-immunoprecipitated proteins. M: Protein marker; 1: Positive sample of His pull-down; 2: Negative control of His pull-down; 3: Positive sample of co-IP; 4: Negative control of co-IP.

Supplementary Figure 2 Bioinformatics analysis of *Et*eIF-5A cDNA and deduced amino acid sequence. Underlined, amidation site; dotted underlined, casein kinase II phosphorylation site; double underlined, N-myristoylation site; yellow background, protein kinase C phosphorylation site; italics, translation initiation factor 5A (eIF5A); wavy underlined, eIF5A hypusine signature; gray background, bacterial Ig-like domain 1 domain profile; boxed, KOW motif; red font, eIF5A hypusine oligonucleotide/oligosaccharide-binding fold motif; asterisk: stop codon.

Supplementary Figure 3 Co-localization of *Et*eIF-5A and *Et*Ub in *Eimeria tenella*. A.B: immature schizont (iSC) at 48 h p.i.

Supplementary Figure 4 Negative control of localization by indirect immunofluorescence. A: Healthy rabbit IgG against Spz by UV; B: Healthy rabbit IgG against Mrz by UV; C: Healthy rabbit IgG against Spz invasion for 2h by UV; D: Healthy rabbit IgG against Spz invasion for 24h by UV; E: Healthy rabbit IgG against Spz in bright; F: Healthy rabbit IgG against Mrz in bright; F: Healthy rabbit IgG against Spz invasion for 2h in bright; H: Healthy rabbit IgG against Spz invasion for 24h in bright.

Supplementary Data Sheet 1 Shotgun analysis of mass spectrum screening results.
