# Supplementary figures and images for: Eimeria tenella Translation Initiation Factor eIF-5A That Interacts With Calcium-Dependent Protein Kinase 4 Is Involved in Host Cell Invasion
Source: Front Cell Infect Microbiol. 2021 Jan 22;10:602049. doi: 10.3389/fcimb.2020.602049 (PMC7862772; doi:10.3389/fcimb.2020.602049)

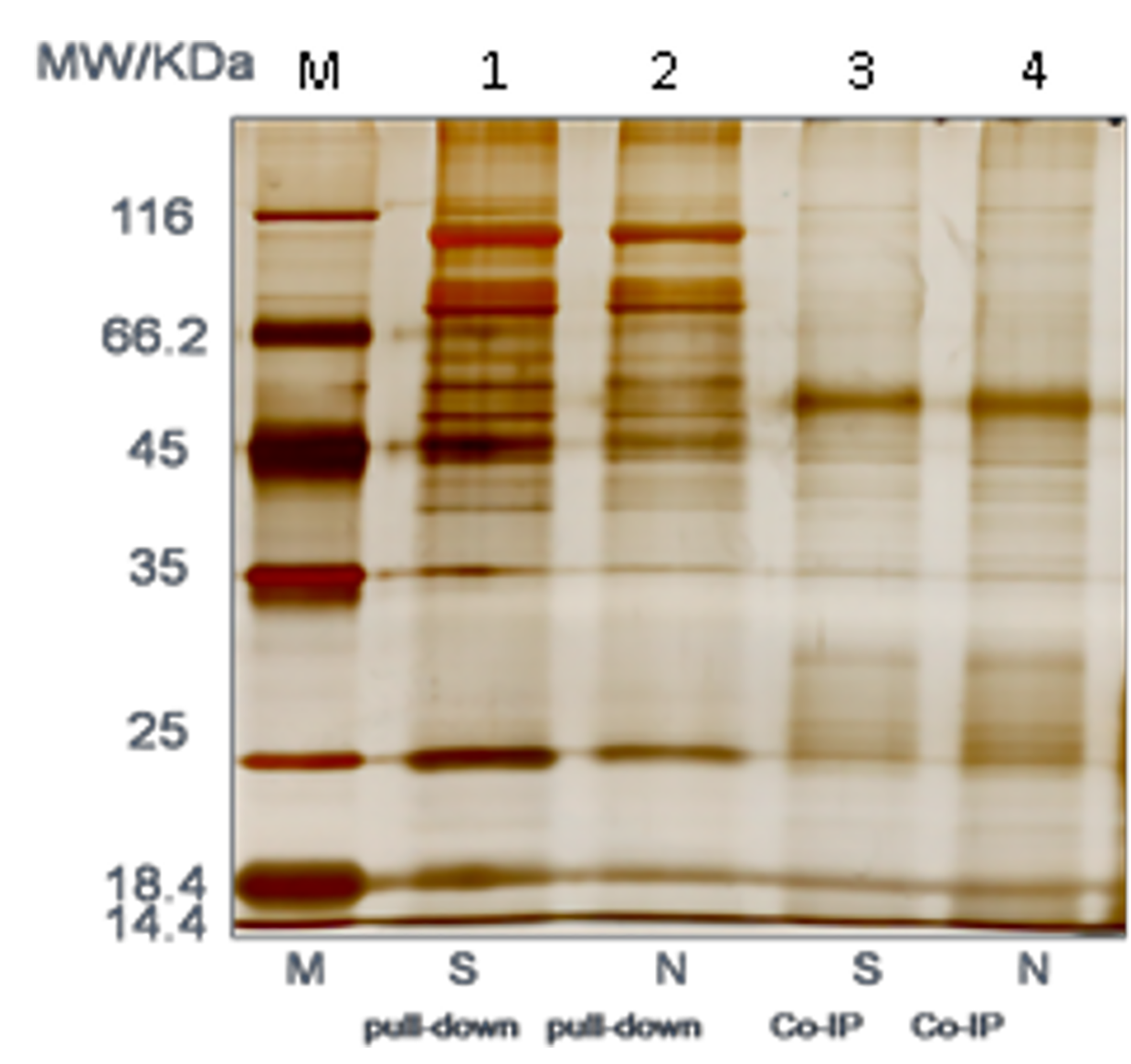

Supplement: Supplementary Figure 1 — SDS-PAGE analysis of pulled-down and co-immunoprecipitated proteins. M: Protein marker; 1: Positive sample of His pull-down; 2: Negative control of His pull-down; 3: Positive sample of co-IP; 4: Negative control of co-IP. [file Image_1.tif]

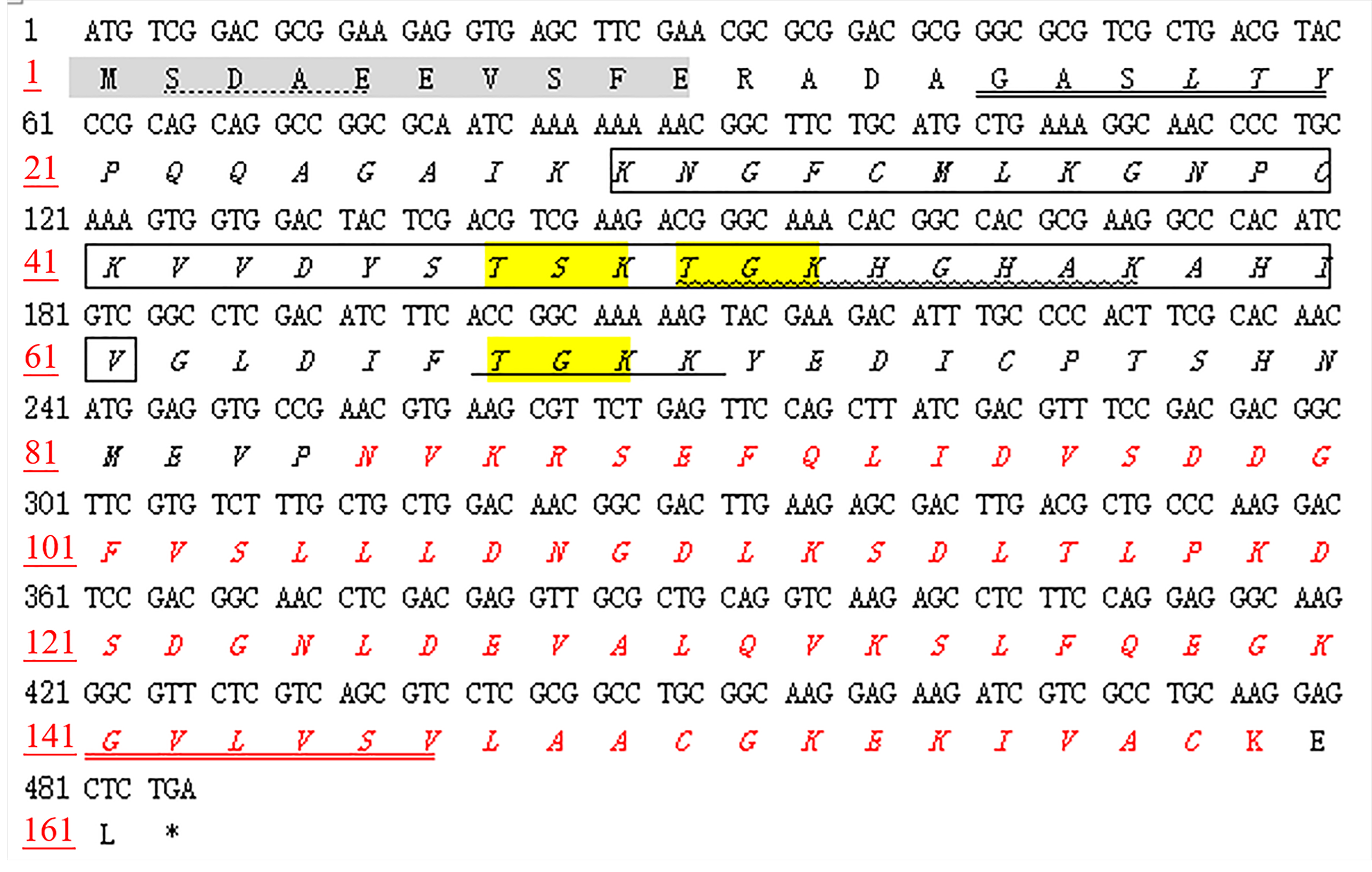

Supplement: Supplementary Figure 2 — Bioinformatics analysis of EteIF-5A cDNA and deduced amino acid sequence. Underlined, amidation site; dotted underlined, casein kinase II phosphorylation site; double underlined, N-myristoylation site; yellow background, protein kinase C phosphorylation site; italics, translation initiation factor 5A (eIF5A); wavy underlined, eIF5A hypusine signature; gray background, bacterial Ig-like domain 1 domain profile; boxed, KOW motif; red font, eIF5A hypusine oligonucleotide/oligosaccharide-binding fold motif; asterisk: stop codon. [file Image_2.tif]

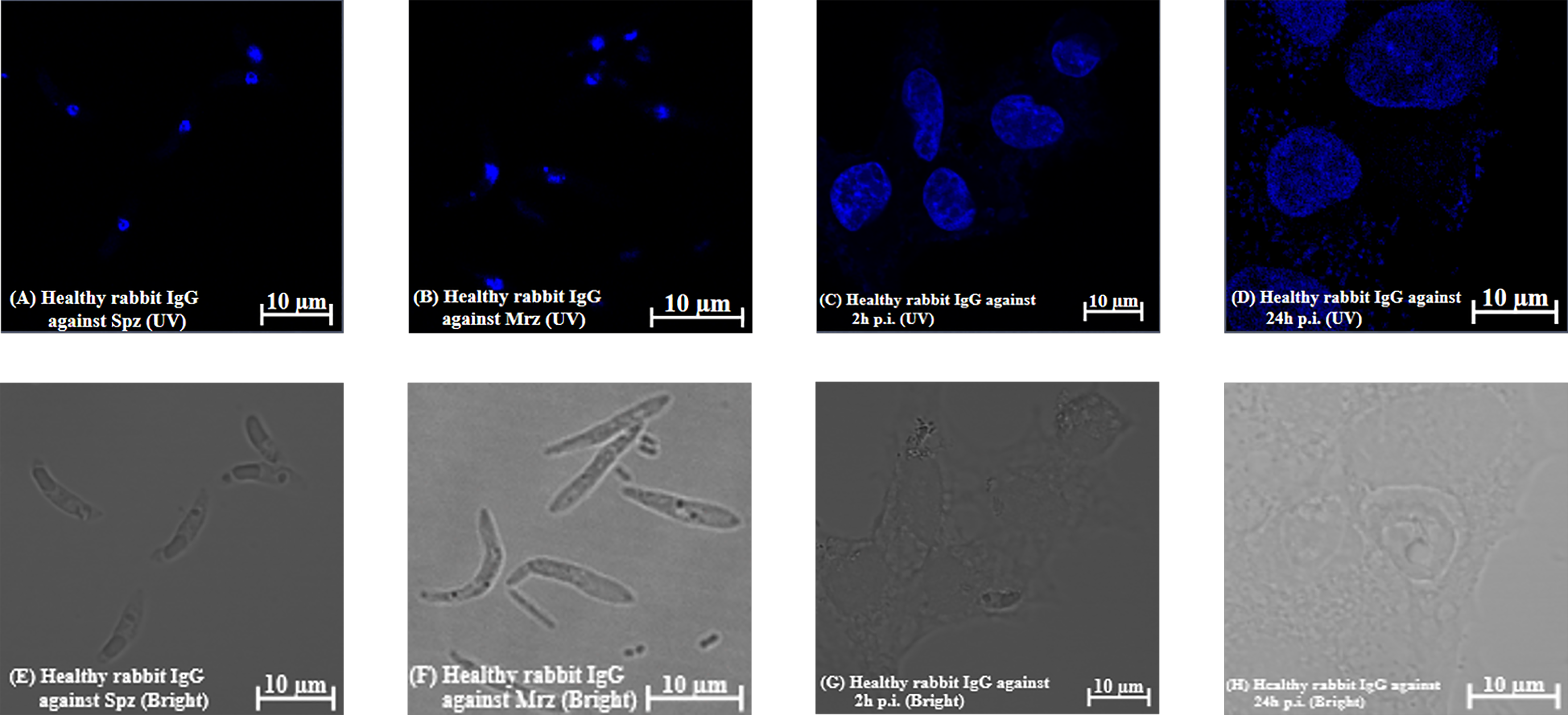

Supplement: Supplementary Figure 3 — Co-localization of EteIF-5A and EtUb in Eimeria tenella. A.B: immature schizont (iSC) at 48 h p.i. [file Image_3.tif]

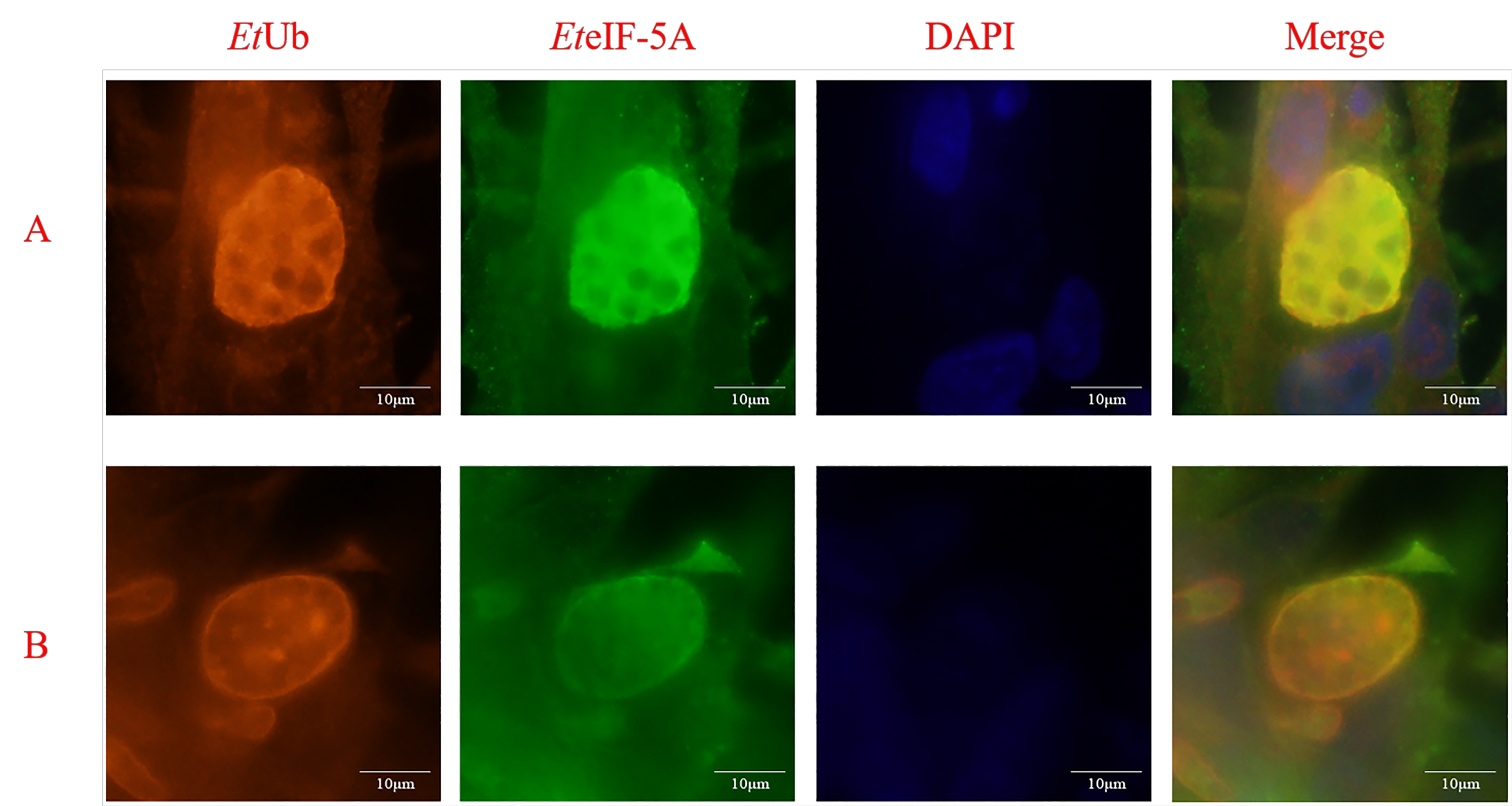

Supplement: Supplementary Figure 4 — Negative control of localization by indirect immunofluorescence. A: Healthy rabbit IgG against Spz by UV; B: Healthy rabbit IgG against Mrz by UV; C: Healthy rabbit IgG against Spz invasion for 2h by UV; D: Healthy rabbit IgG against Spz invasion for 24h by UV; E: Healthy rabbit IgG against Spz in bright; F: Healthy rabbit IgG against Mrz in bright; F: Healthy rabbit IgG against Spz invasion for 2h in bright; H: Healthy rabbit IgG against Spz invasion for 24h in bright. [file Image_4.tif]
